# Near-infrared diffuse *in vivo* flow cytometry

**DOI:** 10.1117/1.JBO.27.9.097002

**Published:** 2022-09-16

**Authors:** Joshua Pace, Fernando Ivich, Eric Marple, Mark Niedre

**Affiliations:** a Northeastern University, Department of Bioengineering, Boston, Massachusetts, United States; b EmVision LLC, Loxahatchee, Florida, United States

**Keywords:** diffuse fluorescence, diffuse *in vivo* flow cytometry, near-infrared light, contrast agents

## Abstract

**Significance:**

Diffuse *in vivo* flow cytometry (DiFC) is an emerging technique for enumerating rare fluorescently labeled circulating cells noninvasively in the bloodstream. Thus far, we have reported red and blue-green versions of DiFC. Use of near-infrared (NIR) fluorescent light would in principle allow use of DiFC in deeper tissues and would be compatible with emerging NIR fluorescence molecular contrast agents.

**Aim:**

We describe the design of a NIR-DiFC instrument and demonstrate its use in optical flow phantoms *in vitro* and in mice *in vivo*.

**Approach:**

We developed an improved optical fiber probe design for efficient collection of fluorescence from individual circulating cells and efficient rejection of instrument autofluorescence. We built a NIR-DiFC instrument. We tested this with NIR fluorescent microspheres and cell lines labeled with OTL38 fluorescence contrast agent in a flow phantom model. We also tested NIR-DiFC in nude mice injected intravenously with OTL38-labeled L1210A cells.

**Results:**

NIR-DiFC allowed detection of circulating tumor cells (CTCs) in flow phantoms with mean signal-to-noise ratios (SNRs) of 19 to 32 dB. In mice, fluorescently labeled CTCs were detectable with mean SNR of 26 dB. NIR-DiFC also exhibited orders significantly lower autofluorescence and false-alarm rates than blue-green DiFC.

**Conclusions:**

NIR-DiFC allows use of emerging NIR contrast agents. Our work could pave the way for future use of NIR-DiFC in humans.

## 1 Introduction

In hematogenous cancer metastasis, circulating tumor cells (CTCs) intravasate from the primary tumor into the peripheral blood. A small fraction of these travel to distant organs via the circulatory system and form secondary tumors.^1^^–^^6^ As such, the ability to detect, count, and characterize CTCs and CTC clusters is of potentially great diagnostic value in clinical management of cancer and in the basic study of cancer biology.^7^^–^^9^ Fewer than 1 CTC per mL of peripheral blood may be indicative of metastatic progression.^10^^–^^15^ “Liquid biopsy” is the current gold standard for the study of CTCs, which involves drawing and analyzing small volume (∼mL) peripheral blood samples using *ex vivo* assays such as *CellSearch*.^16^^,^^17^ While powerful, liquid biopsy is poorly suited to measuring dynamic changes in CTC numbers over time.^18^^,^^19^ In addition, because volume of peripheral blood is fractionally very small compared to the total blood volume (<1%), these may provide a poor overall and statistical picture of CTC numbers.^18^^–^^21^

These limitations have motivated development of optical methods to enumerate CTCs directly in circulation *in vivo*.^22^^–^^27^ Our team developed a preclinical laser-induced fluorescence technique called “diffuse *in vivo* flow cytometry” (DiFC).^28^^,^^29^ DiFC uses specially designed optical fiber probes and highly scattered light to sample blood flowing in large vessels in the tail or the hindleg of a mouse. By measuring the signal emitted from individual cells, DiFC allows noninvasive sampling of about 100  μL of blood per minute. We previously used DiFC to study CTC dissemination in preclinical mouse models including a subcutaneous Lewis lung carcinoma (LLC) and multiple myeloma disseminated xenograft model.^30^^,^^31^ Because DiFC is noninvasive, the entire peripheral blood volume of a mouse can be sampled continuously and repeatedly over time.

Our previously reported DiFC instruments were designed to work with either blue-green [e.g., green fluorescent protein (GFP)] or red (e.g., Cy5) fluorophores that are commonly used in preclinical research. Emerging preclinical fluorophores and fluorescent proteins operates in the near-infrared (NIR) range. Because of the fundamental nature of light transport in biological tissue, NIR light undergoes less attenuation and generation of autofluorescence,^32^ and as such would theoretically be advantageous for DiFC. In fact, we recently showed that NIR light would in principle enable detection of well-labeled CTCs in large blood vessels below the surface with higher signal-to-noise-ratio (SNR) and detection depth than visible light.^33^

Related to this, another long-term goal of our research is potential human translation of DiFC technology.^34^ Nearly, all clinical fluorescence contrast agents operate in the NIR region due to light transport effects. Likewise, in principle, NIR-DiFC would be more suitable for larger limbs and deeper-seated blood vessels, e.g., in the human wrist or forearm.^33^^,^^35^

Motivated by these factors, in this paper, we describe the design and construction of a new NIR-DiFC instrument. Noting that NIR fluorophores typically have lower fluorescence quantum yield than visible fluorophores, we also redesigned our fiber probe assemblies (compared to our previously reported designs^29^) to allow improved geometric light collection and rejection of instrument autofluorescence.

We show proof of concept testing with cells labeled with a NIR fluorescent molecular probe OTL38, which targets folate receptor (FR) over-expressing cells. We show that OTL38-labeled cells are detectable in a tissue simulating flow phantom *in vitro* and in mice *in vivo*.

## 2 Materials and Methods

### 2.1 NIR-DiFC Instrument

**Fig. 1:**
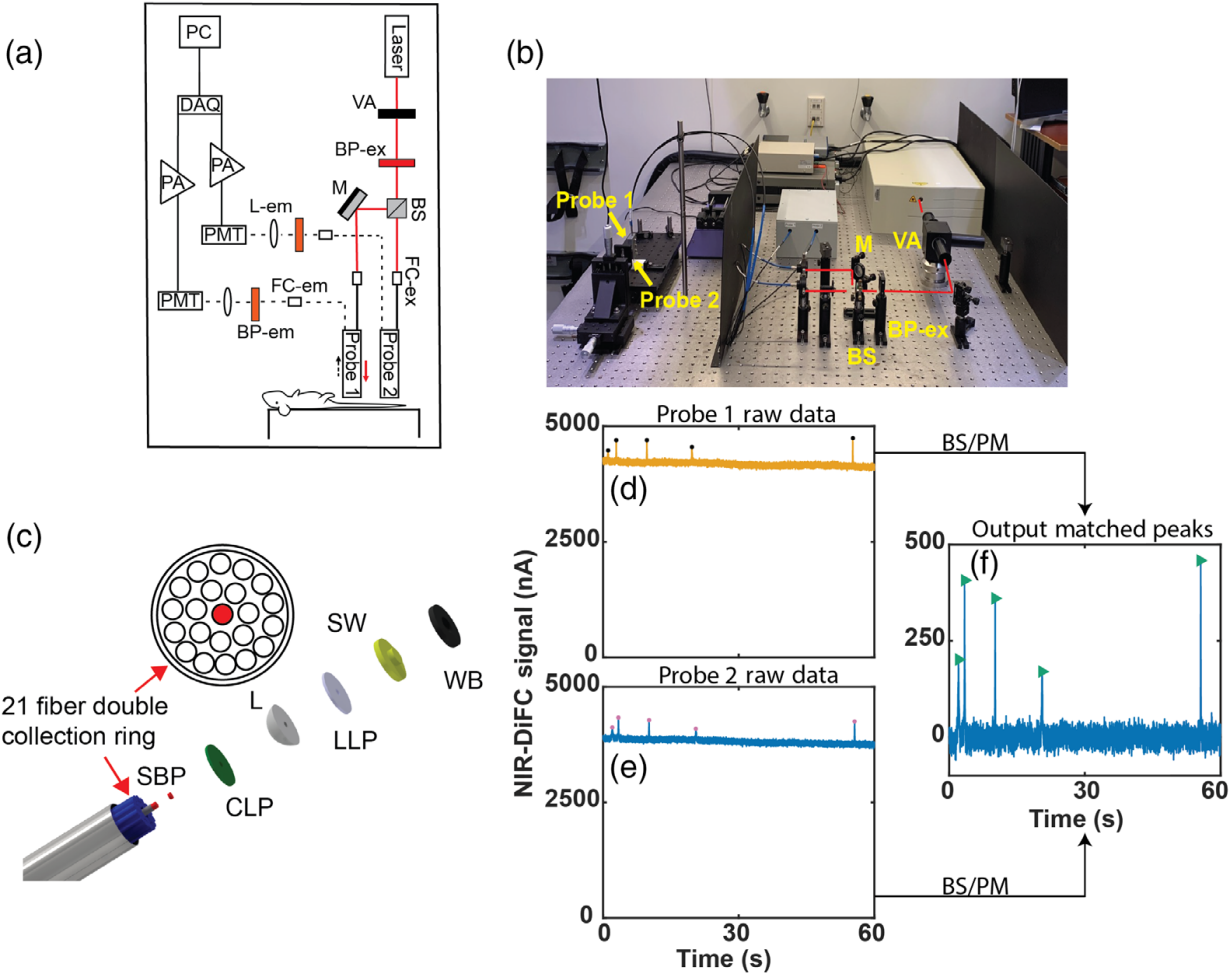
The NIR-DiFC instrument: (a) optical schematic and (b) photograph with main components labeled. (c) Schematic of the newly designed NIR-DiFC fiber probe bundle; see main text for component details. (d), (e) Representative NIR-DiFC data measured from probes 1 and 2, respectively, before postprocessing. As discussed in detail in the text, this requires background subtraction (BS) and peak matching (PM). (f) Postprocessed data after BS and identification of forward-matched peak (green arrowheads) between the two probes.

The schematic of NIR-DiFC is shown in Fig. 1(a). The light source is a tunable pulsed laser (Mai Tai XF-1, Spectra Physics, Santa Clara, California) with excitation wavelength set to 770 nm. The power is adjusted with a variable attenuator before it is passed through a 766/13  nm bandpass clean up filter (BP-ex; FF01-766/13-25, IDEX Health and Science LLC, Rochester, New York). The light is then split into two beams with a beam splitter (BS; 49005, Edmund Optics, Barrington, New Jersey) before being coupled with a collimation package (FC-ex; F240SMA-780, Thorlabs Inc., Newton, New Jersey) into source fibers of the fiber probe assemblies (see Sec. 2.2). The light power at the sample is set to 25 mW. The output of the probe collection fibers is collimated (FC-em; F240SMA-780, Thorlabs), and the light is passed through an 810/10 nm bandpass emission filter (BP-em; FF01-810/10-25, IDEX Health and Science LLC) before being focused on to the surface of a photomultiplier tube (PMT) (H10721-20, Hamamatsu, Bridgewater, New Jersey) with a 30-mm focal length lens (L-em; 67543, Edmund Optics). The PMTs are powered by a power supply (C10709; Hamamatsu). Output signals from the PMTs are filtered with an electronic 100 Hz low-pass filter, amplified with a low-noise current preamplifier (SR570, Stanford Research Systems, Sunnyvale, California), and then acquired with a data acquisition board (USB-6343 BNC; National Instruments, Austin, Texas). Figure 1(b) shows a photograph of the instrument.

### 2.2 Integrated NIR-DiFC Fiber Probe Design

NIR-DiFC uses custom-designed integrated fiber probe assemblies as shown in Fig. 1(c) (EmVision LLC, Loxahatchee, Florida). The design is an improvement compared to our previous fiber probe design^29^ with better geometric collection efficiency and autofluorescence suppression. Each probe is constructed with 21 all silica low hydroxyl (OH) content 300  μm core 0.22 NA collection fibers, arranged in a 7-fiber inner ring and 14-fiber outer ring. The 21 collection fibers are arranged around a single fiber for laser delivery, which is also all silica 300-μm core low OH, 0.22 NA fiber.

A donut-shaped 807-nm long-pass filter (LLP; BLP01-785R, IDEX Health and Science LLC) is positioned in front of the 21 collection fibers to reject laser light and pass collected fluorescence light from the sample. The source delivery fiber has a smaller 709/167  nm bandpass filter (SBP; FF01-709/167, IDEX Health and Science LLC) positioned in front, which allows the light to pass but rejects any other interfering light created from autofluorescence of the probe materials. The distal end of the probe also has a converging sapphire lens (L) with a hole drilled in the center and a second lens LLP with a hole in the center placed on the lens. The laser fiber passes through the center hole of the fiber filter, lens, and lens filter, which eliminates any reflected laser light from the lens surface that can create undesired autofluorescence from the probe materials. The LLP filter further reduces the laser light, which can otherwise back reflect from the sample into the probe and create unwanted background fluorescence. A magnesium fluoride stepped window (SW) is attached to the lens, lens filter, and excitation fiber assembly. This SW allows the laser light and collection light paths to overlap at the distal window tip-sample interface, maximizing collection of fluorescent light. The SW and window block reject undesired light from being collected that is excited outside of the desired sample region and improves blocking efficiency of the long-pass filters. The fibers, lens, and other optical components are placed inside a 2.4-mm outside diameter stainless steel needle tube.

### 2.3 Fluorescent Microspheres

We used “Jade Green Low Intensity” (JGLI; FL100782, Spherotech, Lake Forest, Illinois) NIR fluorescent microspheres. These microspheres are ∼10 to 14  μm in diameter and (as we show) have similar fluorescence brightness to CTCs labeled with an NIR fluorophore.

### 2.4 NIR-DiFC Testing in Tissue-Mimicking Flow Phantoms *In Vitro*

**Fig. 2:**
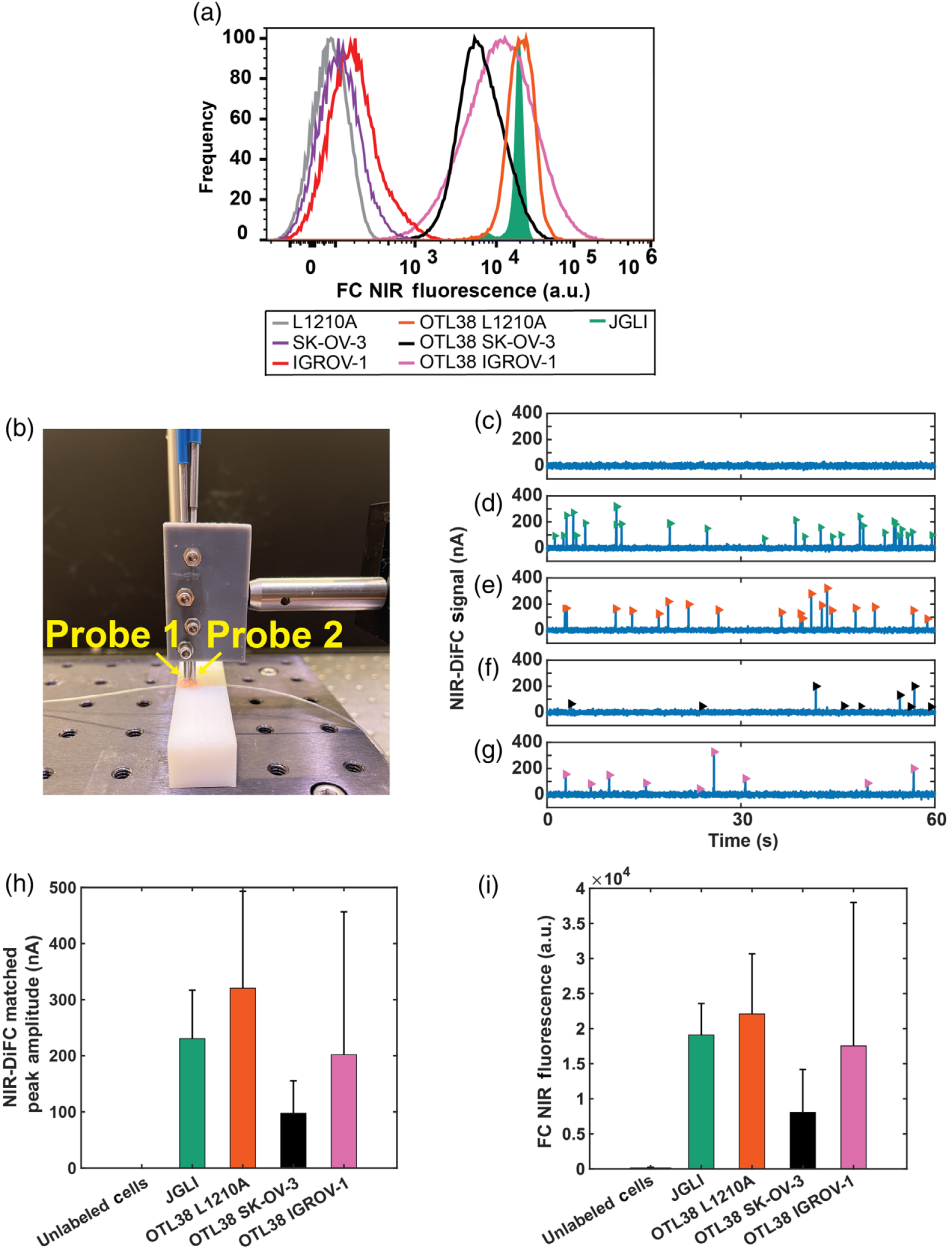
(a) The brightness of OTL38-labeled FR+ cells and JGLI microspheres with flow cytometry. (b) NIR-DIFC with a tissue mimicking flow phantom model. Representative NIR-DiFC data are shown after signal processing for (c) control suspensions of unlabeled L1210A cells, showing no false positive counts, (d) JGLI microspheres, OTL38-labeled (e) L1210A cells, (f) SKOV-3 cells, and (g) IGROV-1 cells. Each peak (arrowhead symbols) represents a forward-matched detection. (h) Comparison of the mean amplitude (intensity) of detected peaks for each sample measured with NIR-DiFC, compared to that measured using (i) flow cytometry, showing good general agreement with NIR-DiFC fluorescence measurements.

NIR-DiFC was first tested using a tissue-mimicking flow phantom [Fig. 2(a)] as we have used previously.^18^^,^^28^^,^^30^^,^^31^^,^^36^ The phantom is made from high-density polyethylene block and has similar absorption and scattering properties to biological tissue.^18^ Microbore Tygon tubing (TGY1010C, Small Parts, Inc., Seattle, Washington) was inserted into a hole drilled into the phantom at a depth of 0.75 mm. This depth was chosen to match the depth of the ventral caudal artery in the mouse tail.^37^ Using a microsyringe pump (702209, Harvard Apparatus, Holliston, Massachusetts), 1 mL suspensions of microspheres or NIR fluorophore labeled cells (see Sec. 2.5) suspended in PBS at a concentration of $10^{3}\;\mathrm{per}\;\mathrm{mL}$ were flowed through the phantom at a flow rate of 50  μL/min.

### 2.5 NIR Fluorescent Labeling of Cells with OTL38

We used L1210A immortalized murine leukemia cells, which were previously modified to over-express FR.^38^^–^^40^ We also used SK-OV-3 (Angio-Proteomie, Boston, Massachusetts) and IGROV-1 (Sigma Aldrich, St. Louis, Missouri), which are both naturally expressing FR+ immortalized human ovarian cancer cell lines. All cells were cultured in RPMI 1640 folic acid deficient media (Gibco 27016021; ThermoFisher Scientific, Waltham, Massachusetts) supplemented with 10% fetal bovine serum (Gibco 16000044; ThermoFisher Scientific) and 1% penicillin/streptomycin (Gibco 15140122; ThermoFisher Scientific) and incubated at 37°C with 5% ${\mathrm{CO}}_{2}$.

OTL38 (On Target Laboratories, West Lafayette, IN) is a FRα-targeting fluorescent small-molecule (MW 1414.42 Da) contrast agent that has previously been characterized in addition to recently being fully FDA approved for the use in fluorescence guided surgery.^41^^–^^43^ OTL38 is a conjugate between a folate analog and S0456 dye [similar fluorescence spectrum to indocyanine green (ICG)] with a maximum excitation wavelength of 776 nm and stokes shift of 17 nm.^44^

1 mL suspensions of $10^{6}$ FR-over-expressing L1210A, SK-OV-3, or IGROV-1 cells in 2% FBS in PBS (Gibco 10010049; ThermoFisher Scientific) were coincubated with 200 nM (20  μL of 10  μM stock) OTL38 at 37°C with 5% ${\mathrm{CO}}_{2}$ for 1 h. Cells were then washed twice with PBS before start of experiments.

### 2.6 Flow Cytometry

OTL38 was analyzed using a benchtop Attune NXT flow cytometer (FC) (ThermoFisher Scientific). NIR fluorescence was collected using a 637-nm excitation wavelength and a 780/60-nm emission filter. CellTrace CFSE fluorescence was collected using a 488-nm excitation wavelength and 530/30-nm emission filter

### 2.7 Validation of NIR-DiFC in Mice *In Vivo*

All mice were handled in accordance with Northeastern University’s Institutional Animal Care and Use Committee (IACUC) policies on animal care. Animal experiments were carried out under Northeastern University IACUC protocol #21-0412R. All experiments and methods were performed with approval from and in accordance with relevant guidelines and regulations of Northeastern University IACUC.

For proof-of-concept testing of NIR-DiFC *in vivo*, we injected L1210A cells intravenously in nude mice.^21^^,^^36^ $10^{6}$ L1210A cells prelabeled with OTL38 *in vitro* as above were suspended in 100  μL of cell culture media and were injected intravenously via the tail vein (N=3) of 6- to 8-week-old female Athymic nude mice (Athymic NCR Nu/Nu/553; Jackson Laboratory, Bar Harbor, Maine). NIR-DiFC was preformed 10 min after injection on the ventral caudal tail artery for 60 min.

In additional experiments (N=3 mice), we double-stained the L1210A cells with OTL38 and CellTrace CFSE green dye (Invitrogen C34554; ThermoFisher Scientific) prior to injection. This allowed us to count L1210A cells in blood samples removed from mice following NIR-DiFC (see Sec. 3) by identification of the double-labeled population. 1 mL suspensions of $10^{6}$ FR-over-expressing L1210A in PBS were coincubated with 5 mM CFSE in the dark at 37°C with 5% ${\mathrm{CO}}_{2}$ for 20 min. Then 5  μL of complete culture media was added, and the suspension was returned to the dark 37°C with 5% ${\mathrm{CO}}_{2}$ incubator for 5 more minutes. The cells were then resuspended in complete media for 10 min before OTL38 labeling. Following NIR-DIFC scanning, blood draws were performed by terminal cardiac puncture. Blood was prepared for analysis by the benchtop flow cytometry by adding it to 2 mL 10× RBC Lysis buffer (420301; BioLegend, San Diego, California) diluted to 1× in 18 mL sterile water for 15 min. Suspensions were then washed twice with PBS and resuspended to a final concentration of 3 mL.

Although *in vivo* labeling of CTCs is beyond the scope of this paper, we performed an additional set of experiments to study how the presence of free OTL38 in circulation could affect background NIR-DiFC signal and noise. To investigate this effect, we injected 2.5  μg OTL38 in 100  μL PBS intravenously via the tail vein (N=3) of 6- to 8-week-old female Athymic nude mice. Each mouse was then scanned with NIR-DiFC 3-, 6-, 9-, 12-, and 24-h postinjection. The noise calculated at each point was then used to estimate the effect on the SNR and detectability of labeled cells as discussed below.

### 2.8 DiFC Data Analysis

During NIR-DiFC scanning, data are recorded continuously with a computer separately on both fiber probes [Figs. 1(d) and 1(e)]. We used the DiFC postsignal processing approach reported in detail by us previously,^28^ which in brief used the following steps.

1. Subtraction of the signal background. This is done by subtraction of the signal moving median value with 5-s window.
2. Calculation of the signal noise (standard deviation) postbackground subtraction (BS) over a 1-min moving window.
3. Identification of peak candidates with a threshold above five times the local noise, which gives a minimum SNR of 20 log 10(5)=13.9  dB.

In addition, use of two DiFC probes allows us to apply an additional matching condition [Fig. 1(f)] for detections. Peak candidates from the probes are matched in either the forward or reverse direction based on the peak amplitude, width, and transit time between the detectors. Peaks that are not matched are discarded from the analysis. This ensures that detected peaks are from cells moving in target arteries or veins. Unmatched peaks were normally from detected cells in the capillary bed or other small blood vessels. This also discards spurious signals due to instrument noise or motion artifacts, which normally appear as coincident peaks on both probes. Endogenous autofluorescence from biological tissue is generally constant over the timescale of seconds (as opposed to transient fluorescent peaks from CTCs). Hence, these contribute to the background signal and are subtracted off in step 1 above.

## 3 Results

### 3.1 NIR-DiFC Detection of Microspheres and Cells in Flow Phantom *In Vitro*

We first performed *in vitro* testing of NIR-DiFC by labeling several FR positive cell lines with OTL38. We verified labeling using FC as shown in Fig. 2(a). Differing peak amplitudes were due primarily to the variations in OTL38 contrast agent uptake by the cells and varying levels of FR expression in the different cell lines. We then tested NIR-DiFC using an optical flow phantom model [Fig. 2(b)], which, as previously shown, approximates the optical properties of bulk biological tissue in the NIR range.^33^ We passed suspensions of fluorescent microspheres and OTL38-labeled cells through the phantom to mimic cells flowing through a blood vessel. Peaks were never observed in control solutions of either PBS alone or unlabeled cells. An example NIR-DiFC data scan for unlabeled L1210A cells is shown in Fig 2(c). Representative NIR-DiFC data (background subtracted) are shown in Figs. 2(d)–2(g) for JGLI microspheres, and OTL38-labeled L1210A cells, SKOV-3 cells, and IGROV-1 cells, respectively. In each plot, every vertical peak represents a forward-matched detection between the two NIR-DIFC probes.

The mean peak amplitude (fluorescent intensity) measured with NIR-DiFC for the different microspheres and cell types averaged over a minimum of 90 peaks is summarized in Fig. 2(h). As shown, the relative detection intensities are approximately in agreement with those measured with FC as shown in Fig. 2(i), with similar variability in cell brightness (due to FR expression and OTL38 binding) observed.

Although we used suspensions of $10^{3}$ cells per mL here, as we and others have shown previously^28^^,^^31^^,^^36^ in mouse models of metastasis, we expect in the range 10 to 100 CTCs per mL of peripheral blood, corresponding to an expected *in vivo* DiFC count rate (assuming ∼100  μL blood flow per minute in the mouse tail^28^^,^^31^^,^^36^) of ∼60 to 600 cells per hour.

### 3.2 *In Vivo* Validation of NIR-DiFC with OTL38-Labeled L1210A Cells

**Fig. 3:**
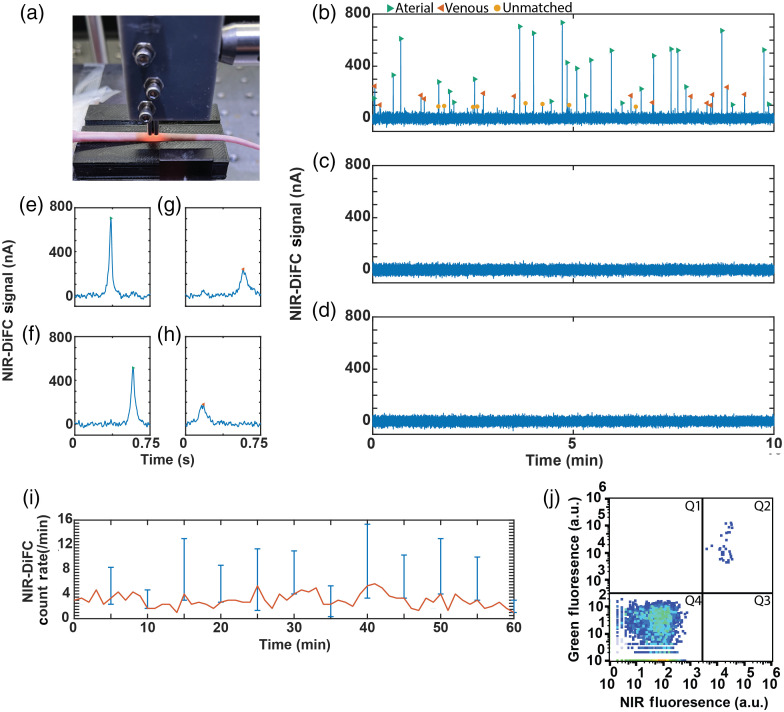
*In vivo* testing of NIR-DiFC. (a) Fiber probes were placed on the surface of the mouse tail approximately above the ventral caudal artery. (b) Representative NIR-DiFC data measured from a mouse injected intravenously with OTL38-labeled L1210A cells. Each green arrowhead is an arterial matched cell (traveling in artery), each orange arrowhead is a venous matched cell (traveling in vein), and each yellow-labeled peak represents a detected unmatched cell (traveling in capillary). (c) Representative NIR-DiFC data from a control mouse injected with L1210A. (d) Representative NIR-DiFC data from an uninjected control mouse. (e), (f) Cell detected sequentially in probes 1 and 2, indicating a cell traveling in the arterial direction. (g), (h) Cell detected sequentially in probes 2 and 1, indicating a cell traveling in the venous direction. (i) The mean cell detection rate over a 1-h scan indicating that cell numbers were approximately stable in circulation. Range bars show the minimum and maximum values (N=3). (j) We counted L1210A cells still in circulation following NIR-DiFC. Representative flow cytometry data from blood samples. As described in the text, L1210A cells were prelabeled with both CFSE (green) and OTL38 (NIR) prior to injection appearing as the double-labeled population (Q2).

We next tested NIR-DiFC in nude mice *in vivo*. L1210A cells were prelabeled with OTL38 *in vitro* and then injected intravenously via the tail vein. The fiber probes were placed on the skin surface on the ventral side of the tail, approximately above the ventral caudal vascular bundle as shown in Fig. 3(a). Representative data from an OTL38-L1210A injected mouse are shown in Fig. 3(b). These peaks were never observed in control mice injected with unlabeled L1210A cells [Fig. 3(c)] or in control (uninjected) mice as shown in Fig. 3(d).

As described in Sec. 2.8, for *in vivo* measurements, we imposed a matching condition, examples of which are shown in Figs. 3(e)–3(h). Cells traveling in the forward, arterial direction are detected as peaks sequentially measured on the first [Fig. 3(e)] and then second [Fig. 3(f)] probes and separated by a time delay due to the 3-mm separation between probes. These are indicated as green forward arrow markers in the longer data trace in Fig. 3(b). Likewise, cells traveling in the reverse (venous) direction are sequentially detected by the second and then first probe [Figs. 3(g) and 3(h)] and are indicated as orange reverse arrows. As we noted previously, cells moving in the arterial direction (forward) are generally faster moving (shorter period between sequential detections) than those moving in the venous direction (reverse).^28^ Unmatched peaks—likely due to cells traveling in the network of capillaries—are indicated as round markers.

As shown in Fig. 3(i), the cell detection count rate stayed relatively constant over 60-min scans, which is consistent with our previously observed circulation kinetics in the same mouse model and cell line.^36^ Although we injected $10^{6}$ L1210A cells, as in our prior work,^36^ many are cleared from circulation in the “first pass effect”^45^ in the liver and lungs, after which a relatively small number remain in circulation. To show this, in additional experiments (N=3 mice), we drew ∼1  mL blood after NIR-DiFC scanning and used FC to count L1210A cells in the blood [Fig. 3(j)]. From these, we found on average 29±10  cells/mL of peripheral blood. The average measured average NIR-DiFC count rate in the last 10 min of the scan was 2.2±0.9  cells/min. Assuming a typical blood flow rate in the tail ventral caudal artery of 100  μL/min,^46^ this corresponds to ∼22±9  cells/mL in the blood, showing very good agreement between the methods.

### 3.3 Free OTL38 in Circulation Increases the Background Noise and Affects Cell Detectability

**Fig. 4:**
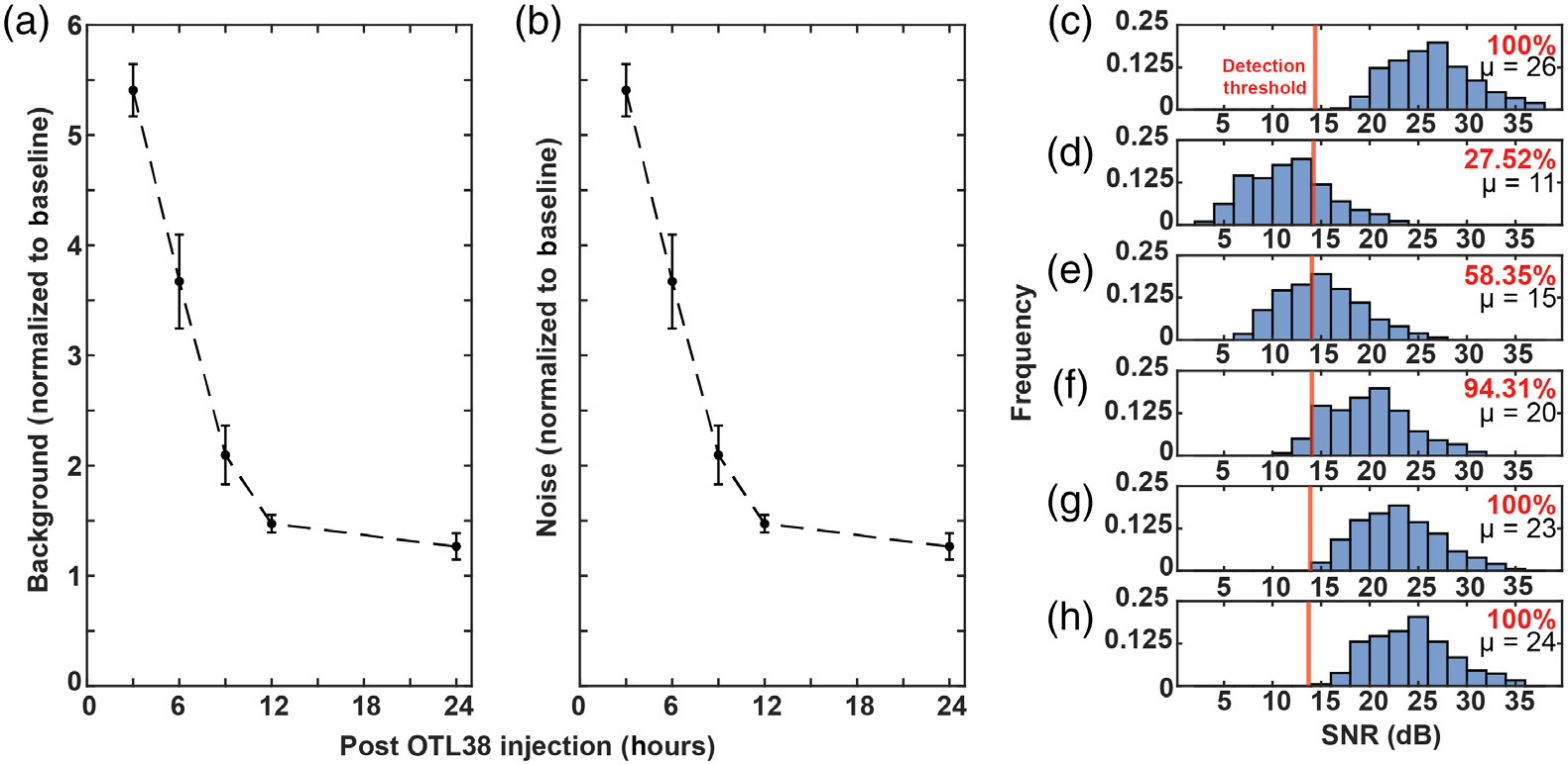
We studied the effect of injection of 2.5  μg OTL38 on the background NIR-DiFC signal and noise following injection. (a) The background increased by a factor of ∼5.5, 3 h post OTL38 injection compared to the preinjection. By 24 h, the background was near baseline. (b) Likewise the noise (standard deviation of the background), followed a similar trend and returned close to baseline by 24 h. Considering the (c) SNR of detected OTL38-labeled L1210A cells as in Fig. 3, we estimated the effect of the increased noise on the SNR (d) 3 h, (e) 6 h, (f) 9 h, (g) 12 h, and (h) 24 h post-OTL38 injection. The red vertical line at 13.9 dB represents the cell detection threshold, and the percentage of detectable cells compared to baseline as well as the mean SNR is indicated on each plot.

In the future, we plan to use OTL38 as an injectable molecularly targeted fluorescent contrast agent (as opposed to prelabeling cells as we have done here).^36^ Free dye in circulation increases NIR-DiFC background noise and hence may affect cell detectability. To study this, we injected 2.5  μg of free OTL38 and measured the subsequent NIR-DiFC background signal. As shown in Fig. 4(a), at 3 h, the background increased by a factor of ∼5.5 compared to preinjection baseline background. As the OTL38 cleared from circulation, the background decreased over time and was close to baseline after 24 h. Likewise, the noise (standard deviation) on the background exhibited a similar clearance trend as shown in Fig. 4(b). To understand the effect of this noise increase on cell detectability, we considered the distribution of detected SNRs of OTL38-labeled L1210A cells (from the studies in Fig. 3) as shown in Fig. 4(c). Given the noise levels measured at different timepoints in Fig. 4(b), we calculated the resulting distribution of SNRs for 3-, 6-, 9-, 12-, and 24-h postinjection in Figs. 4(d)–4(h), respectively. The vertical red line in each panel indicates the 13.9-dB detection threshold. As shown, increased noise at earlier timepoints (3- to 9-h postinjection) would reduce the fraction of cells detected compared to baseline. However, by 12 h, the noise returned to sufficiently low levels, suggesting sufficient clearance that free probe would not affect detectability. This is a subject of ongoing work in our group.

We also note that the mean peak SNR at baseline background [Fig. 4(c)] was 26 dB. We previously used a green folate targeted molecular probe (EC17) with the same mouse model and found a mean peak SNR of 18 dB (at baseline background, no free EC17 intravenously injection).^36^ Although there were differences in the instrument, fluorophores, and placement of the probe (tail versus hindleg), the SNR was significantly improved compared to our blue-green (b-DiFC) system.

### 3.4 NIR-DiFC Autofluorescence is Significantly Lower than Visible DiFC

**Fig. 5:**
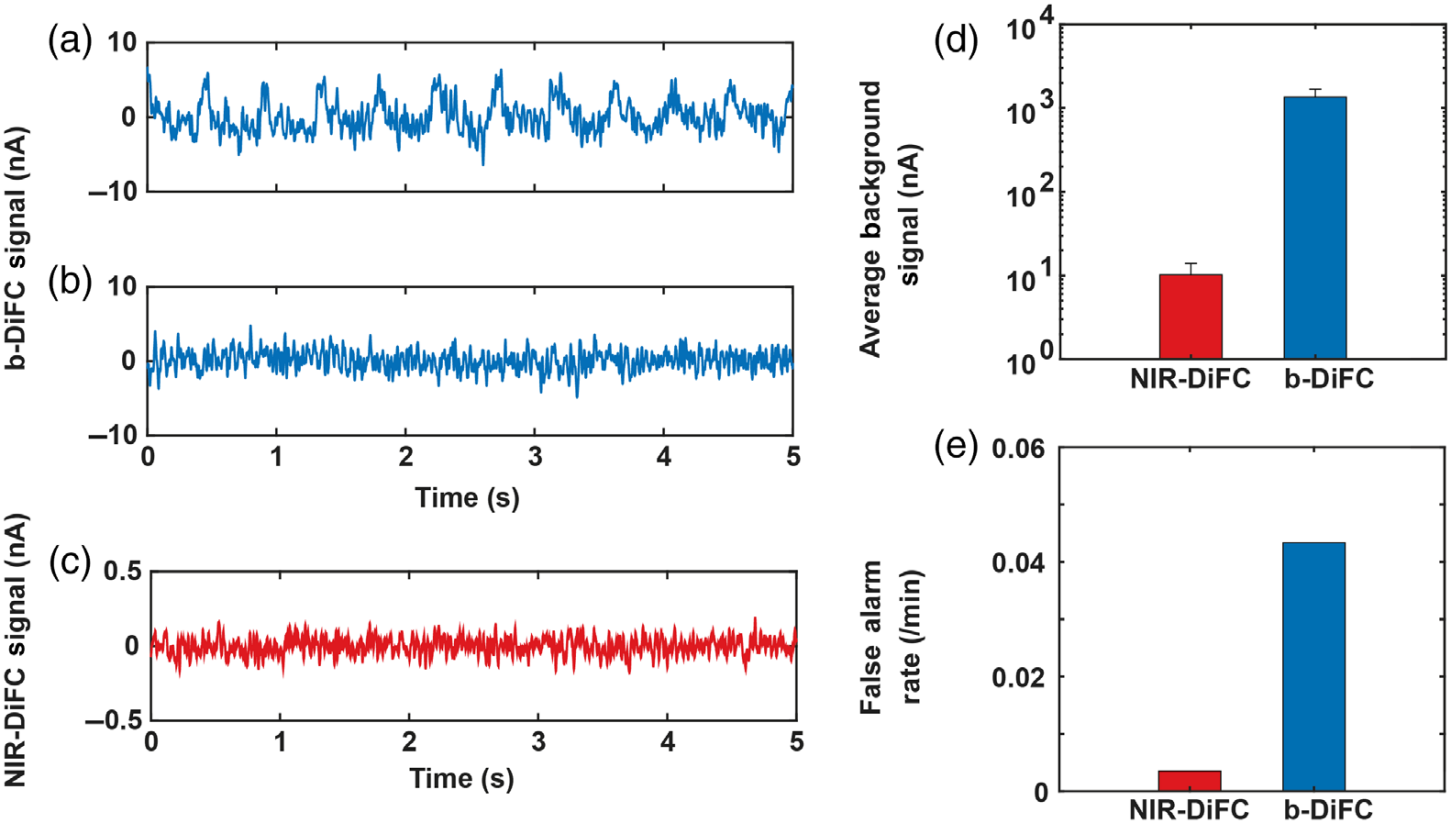
(a) Example breathing artifacts measured with our blue-green b-DiFC instrument. (b) These generally can be corrected by simply adjusting the b-DiFC probe position on the skin, although this causes loss of time. (c) In contrast, these motion (breathing) artifacts were not observed with NIR-DiFC measurements. (d) Use of NIR laser light for DiFC resulted in significantly lower tissue autofluorescence than blue light. (e) In practice, this resulted in a reduction in DiFC FAR.

In addition to the advantages of NIR-DiFC described above, as summarized in Fig. 5, we observed that tissue autofluorescence is markedly reduced and the signal is free of motion artifacts^33^ compared to our preclinical GFP-compatible b-DiFC instrument.^31^ Use of blue laser light is widely understood to yield higher biological tissue autofluorescence than NIR light.^47^ Likewise, use of blue-green light in fluorescence measurements is in general more sensitive to breathing (motion) or photoplethysmography artifacts, which has been attributed to sensitivity of the measurement to volume expansion and contraction in the capillary bed.^48^^–^^50^ We occasionally observed this in our prior work with b-DiFC: an example b-DiFC data trace showing a breathing artifact is shown in Fig. 5(a).^18^^,^^30^ Generally, when breathing artifacts are observed, we simply adjust or reposition the b-DiFC fiber probe on the mouse skin surface to remove it [Fig. 5(b)]. However, these artifacts may occasionally result in false-positive peak detections.

In contrast, these motion artifacts were not observed in NIR-DiFC [e.g., Fig. 5(c)], which we attribute to lower sensitivity to motion artifacts and generally reduced levels of tissue autofluorescence.^32^ This is illustrated in Fig. 5(d), which shows the mean tissue autofluorescence for b-DiFC and NIR-DiFC systems. The laser power at the surface was 20 and 25 mW for the b-DiFC and NIR-DiFC, respectively. We note that in making this comparison, we explicitly standardized (using the Hamamatsu H10722-20 PMT specifications documentation) the output current between the two systems correcting for voltage output (b-DiFC) and current output (NIR-DiFC) versions of the PMT module and correcting for differences in control voltage applied between PMTs (which were lower with the b-DiFC system than NIR-DiFC system). In practice, as shown in Fig. 5(e), lower noise levels resulted in a lower false alarm rate (FAR) with the NIR-DiFC system compared to the b-DiFC system.

## 4 Discussion and Conclusions

We previously reported red and blue-green versions of DiFC, which we used to enumerate fluorescently labeled or fluorescent protein expressing CTCs during metastasis development in several mouse models.^18^^,^^30^^,^^31^ Beyond small animal models, one long-term goal for DiFC is potential use in humans.^34^ As a first step to this, here we reported development of a NIR version of DiFC. The rationale was threefold. First, we showed previously that NIR-DiFC should (in principle) permit detection of CTCs in blood vessels 2 to 4 mm deep.^33^ Hence, major blood vessels in the wrist or forearm of a human should be accessible with NIR-DiFC.^35^ The blood flow rates in such vessels are in the order of 100  mL/min, significantly higher than blood flow rates in mice, and suitable for sampling large peripheral blood volumes relatively short (∼10  min) DiFC scans.^34^

Second, use of NIR laser light for DiFC yields lower tissue autofluorescence and lower incidence of motion artifacts in DiFC data (Fig. 5). In practice, this results in fewer false positive CTC detections and obviates the need for occasional realigning of the instrument. NIR fluorophores are typically less bright than visible fluorophores, but as we showed the improved NIR-DiFC fiber probe design allowed efficient geometric collection of fluorescent light. Combined with lower autofluorescence, we showed clear detection of CTCs *in vivo* with SNRs ∼8  dB higher than analogous experiments with our b-DiFC system.^36^

Third, many emerging fluorescence molecular contrast agents use NIR fluorophores because they allow imaging in deeper tissues both in mice preclinically and in humans clinically.^51^ As such, we designed NIR-DiFC to be compatible with these. We are currently evaluating OTL38 for labeling of CTCs for NIR-DiFC. We and others previously showed that small-molecule FR targeted probes have significant potential for bright and specific labeling of CTCs in whole blood.^36^ This is an ongoing area of research in our group.

Related to this, any use of DiFC in humans would also require the use of an administered molecular fluorescence contrast agent that would allow specific and bright labeling of CTCs. We and others have already showed that this is feasible using small molecule or antibody targeted fluorescent molecular probes that are specific to cancer-associated cell surface markers.^36^^,^^52^^,^^53^ There are a large and growing number of molecular contrast agents under development for fluorescence guided surgery with NIR dyes including ICG, S0406, and IRDye80.^51^ In this work, we use OTL38, a folate analog conjugated to S0406, which targets the overexpression of cell surface FR found in many types of cancers including ovarian, non-small-cell lung, mesotheliomas, and triple-negative breast cancers.^54^^,^^55^ Although we only performed NIR-DiFC by injection of OTL38-labeled L1210A cells here, analysis of the cell brightness data in Fig. 2(a) and *in vivo* DiFC measurements in Fig. 4 suggests that more than 90% of IGROV-1 and SK-OV-3 cells also would have been detectable. This is the subject of ongoing work in our lab.

Future work will also evaluate labeling and detection of CTCs by systemic administration of OTL38 *in vivo* (as opposed to prelabeling *in vitro* as we have done here) in preclinical metastasis models. Alternative NIR contrast agents may be tested in the future, as well as use of NIR-DiFC for other types of rare circulating cells.

## Code, Data, and Materials Availability

The datasets generated and analyzed during this work and the MATLAB code are available through the Pennsieve data sharing platform (DOI: 10.26275/amsj-jner). Additionally, MATLAB processing code can be found in a Niedre Lab Github repository: https://github.com/mark-niedre/Design-and-Validation-of-NIR-DiFC (DOI:10.5281/zenodo.6506804).
